# Doct. John N. Frink

**Published:** 1844-12

**Authors:** George G. Brewster


### ARTICLE VI.

*Obituary.* By George G. Brewster, D. D. S., etc.

Departed this life, on Thursday, Oct. 10, at his residence in the city of Portland, Maine, of a pulmonary consumption, Doct. John N. Frink, Æt. 36.

This gentleman was a native of Newington, N. H. In 1829 he established himself in Portland, in the practice of dentistry, with professional accomplishments and facilities acquired in the city of New York, under the tutelage of the well-known dental surgeon, the late Dr. Prentiss. The liberal patronage and marked respect paid to Dr. F. by the community with which he had located himself, elevated his spirits and invigorated him to advance, in a constant and untiring action, in an onward and upward course.

In his improvements in dental practice he discovered that the science of his profession should not be limited to the mere operations on teeth and the formation of artificial substitutes. Important as these parts of art are justly considered in dental practice, they were viewed by him as but secondary in course, and valuable only in proportion to the skill which philosophic intelligence and the result of experience could dictate. The mere manual exercise of the profession, (as is held by a great proportion of the superficial practitioners in operative dentistry,) including all that is requisite, was conceived by him of a too insignificant character to constitute the limits of a dentist in these days of the march of intellect and development of the human physiology.

In his mind, the existing difference between a *dental surgeon* and a mere *operator on teeth*, was involved in an extensive and rightly directed intelligence of the former, and the merely empirical acquisitions of the latter. His sense of honor and of the claims held on him for the skilful performance of his professional duties toward those who should commit themselves to his management, induced him to prepare for a more extensive field of action, and mark out his course for greater usefulness and higher distinction.

With such high-minded views and just ideas of the constituent qualities of dental surgery, Dr. F. felt constrained to devote the leisure times of his office, and even the silent watches of the night, to the acquisition of such knowledge as the exigency of his incompleted acquirements in human physiology might suggest, and the science of dentistry justly demand.

Having had the advantages of an early course of academical exercises, his mind was liberally cultivated for engaging in the pursuit of medical lore. By the preceptoral aid of a doctor in medicine he entered on the study of anatomy and the succeeding branches of medical science. The assiduity with which he applied himself, and the interest felt in the course he was pursuing, rapidly advanced him in intellectual culture.

After having attended through the required courses of lectures in the medical school of Bowdoin College, and taken his part in a *thesis on "Dental Science,"* and an unexceptionably honorable examination before the Faculty, he was reported "*competent*," and the degree of Medicinæ Doctoris was conferred on him.

Dr. Frink did not disappoint his expectation of celebrity; nor did he find his expense of time and money for professional erudition a sacrifice to the shrine of a heartless popularity; nor were self-confidence and the tone to his profession, for his scholastic attainments in medical philosophy, all the requital. So amply versed was Dr. Frink in the *physiology* of the dental organs, general *pathology* and *therapeutics*, that he became celebrated for skill in dental surgery, and was deserving the appellation of Chirurgiæ Dentium Doctoris,—*pro meritis suis.*

Dr. Frink was initiated into the American Society of Dental Surgeons at the annual session held in the city of Philadelphia in 1841, and continued his membership till the time of his decease; during which period he evinced a deeply felt interest in the prosperity of the institution, and ever spoke of that professional fraternity as the power of union, to place intelligent, experienced and skilful practitioners of the dental profession to that elevation and distinction their merits claim, and the candor and gratitude which discriminating beneficiaries will freely acknowledge and gratefully confer.

At our meeting of the American Society of Dental Surgeons, held in the Medical College in Boston in 1842, Dr. Frink gave his constant attendance each day. Since that time his declining health has excluded him from any opportunity of joining with the association, or in any way taking a part in its measures.

Now his works on earth are finished. Death has caused him to retire from the theatre of action, and his seat in our assemblies is vacated. He has gone the way of all flesh, and the living lay it to heart. In the sympathies of those who knew him, especially in the affections of his lonely and disconsolate widow, he lives in the character of his native kindness and various social virtues.

*Excerpt.*—"Dr. Frink for many years was favorably known for talents, skill and integrity in his profession. His long and tedious sickness was characterized by an energy and a self-denying consideration for those around him that will not be forgotten by his friends. Their griefs bear testimony to his social worth in the relations of life which have been thus early broken."—*Portland Argus.*

Portsmouth, N. H., 5 *Fleet street*, *Oct.* 17, 1844.

